# Bioinformatics on the Cloud Computing Platform Azure

**DOI:** 10.1371/journal.pone.0102642

**Published:** 2014-07-22

**Authors:** Hugh P. Shanahan, Anne M. Owen, Andrew P. Harrison

**Affiliations:** 1 Department of Computer Science, Royal Holloway, University of London, Egham, Surrey, United Kingdom; 2 Department of Mathematical Sciences, University of Essex, Wivenhoe Park, Colchester, United Kingdom; 3 Department of Biological Sciences, University of Essex, Wivenhoe Park, Colchester, United Kingdom; National Institute of Environmental and Health Sciences, United States of America

## Abstract

We discuss the applicability of the Microsoft cloud computing platform, Azure, for bioinformatics. We focus on the usability of the resource rather than its performance. We provide an example of how R can be used on Azure to analyse a large amount of microarray expression data deposited at the public database ArrayExpress. We provide a walk through to demonstrate explicitly how Azure can be used to perform these analyses in Appendix S1 and we offer a comparison with a local computation. We note that the use of the Platform as a Service (PaaS) offering of Azure can represent a steep learning curve for bioinformatics developers who will usually have a Linux and scripting language background. On the other hand, the presence of an additional set of libraries makes it easier to deploy software in a parallel (scalable) fashion and explicitly manage such a production run with only a few hundred lines of code, most of which can be incorporated from a template. We propose that this environment is best suited for running stable bioinformatics software by users not involved with its development.

## Introduction

There has been a rapid increase in the number of cloud computing solutions across the computational biology community. For example, cloud computing has already been utilised for bioinformatics workflows [1], comparative genomics [2], gene set analysis for biomarkers [3], identifying epistatic interactions between single-nucleotide polymorphisms [4], microbial sequence analysis [5], multiple sequence alignment algorithms [6], pandemic simulations [7], personal genome variant annotation [8], protein annotation [9], proteomics analysis [10] and systems biology [11].

The community has explored different cloud solutions, such as hybrid clouds [12], [13], Hadoop-like architectures [14], and the Google App Engine [15]. However, despite the wide variety of different cloud computing platforms available, most of the existing work in computational biology has focussed on Amazon Web Services (AWS) as provided by Amazon, in particular their Elastic Cloud Computing (EC2) service [16].

In this paper we will consider an alternative type of cloud computing platform: namely Azure the cloud computing platform provided by Microsoft. The rest of this paper will be organised as follows. We shall briefly explain the qualitative difference between this platform and the more traditional platforms such as Amazon EC2. We will explain the importance of coordinating large numbers of Virtual Machines (VM's) using Job Management software for researchers. We will explain the features of Azure and contrast them with those of other cloud computing platforms, pointing out strengths and weaknesses. We will present results based on a typical bioinformatics workflow using R to analyse microarray data computed on Azure, initially to determine if it reproduces locally computed results and then to determine if its performance is comparable to running the same task locally. Finally we draw conclusions about the applicability of Azure and draw some general lessons on how cloud computing would ideally evolve for bioinformatics.

### Cloud computing fundamentals

There exists an extensive literature providing definitions of cloud computing [17]. We refer the reader to table 1 with cloud computing related definitions. In essence clouds are large server farms which make extensive use of virtualisation to provide outside users with effectively arbitrarily large numbers of Virtual Machines (VM's) and in some respects they can be seen as an extension of the idea of utility computing that was carried forward by Grid Computing in the 1990's [18].

**Table 1:** Definitions of Cloud Computing Terms.

Term	Explanation	Example
VM	Virtual Machine - a piece of software that emulates the behaviour of a separate computer running an Operating System.	
IaaS	Infrastructure as a Service - VM's can be accessed directly via a command line interface.	EC2, RackSpace, OpenStack
PaaS	Platform as a Service - VM's can only be accessed programmatically	Azure, AppEngine, Elastic BeanStalk, Heroku
Job manager	Software which manages the submission of an arbitrary number of executables (jobs) over a large number of computers which typically vary in their parameters. Job Management software will typically include the creation of log files for each run in a systematic fashion and deal with failures in an orderly way.	StarCluster, Generic Worker, Condor, Oracle Grid Engine
Software stack	A set of software that communicate with each other in a hierarchical fashion. In the context of cloud computing, this allows the decoupling of issues that are relevant to each local computer with global issues such as their overall management.	
Image	Bit-for-bit copy of the state of a particular VM which can then be deployed elsewhere. As a result, one can use a VM which runs locally or on a cloud which is configured precisely with the software and data the user requires.	
MapReduce	A protocol for distributed systems that notes that in the analysis of large data sets distributed over many VM's require one (Map) step that has to be executed by all the VM's on the data it has, followed by another (Reduce) step where the results of the Map step are then collated in some fashion to one VM.	Hadoop, HDInsight, Greenplum

The most commonly used cloud computing infrastructures in bioinformatics, such as Amazon EC2, are referred to as Infrastructure as a Service (IaaS), where each VM can be accessed directly via a command line interface. Others, such as Azure, Google AppEngine and Heroku [19] are referred to as Platform as a Service (PaaS) as they supply additional services and programmatic access to each VM. It should be noted that the divisions between these different types of platforms are becoming increasingly blurred - Azure also provides an IaaS and Amazon provides a PaaS built on EC2 called Elastic Beanstalk [20].

Nevertheless there is a substantial difference between using Azure and using IaaS infrastructures which translates into a steep learning curve particularly for bioinformatics developers who typically have a background in writing software for Linux systems.

The above are consistent with formal definitions that are provided by, for example, NIST [21]. It should be noted that these definitions do not necessarily imply that a PaaS is built upon an IaaS. The functional construction of these clouds is complex and beyond the scope of this article.

The basic issues for cloud computing and its application in bioinformatics have already been discussed in detail elsewhere [16], [22]. In brief, the key advantage of cloud computing for bioinformatics researchers is the ability to scale an analysis up and complete the task in as short a period of time as possible. Many bioinformatics researchers do not use high-throughput computing resources to carry out this phase on a frequent basis and hence it is efficient to lease time on a cloud computing platform for a comparatively small sum of money (e.g. hundreds of U.S. dollars) that can be readily absorbed into the day to day costs of a project.

### Batch mode submission

**Figure 1:**
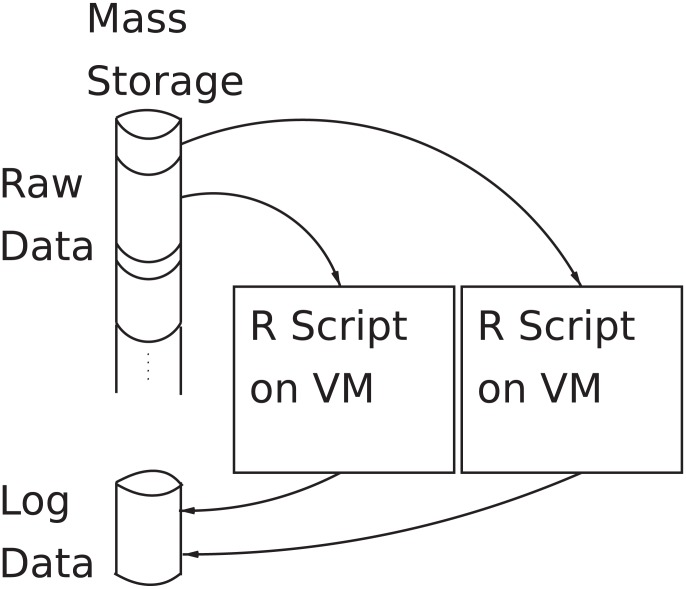
Batch mode operation schematic.

While it is often possible to consider problems which require a high level of parallelisation (using message passing or threading) [23], [24], the prohibitive amount of development time and resource required to do this tends to direct researchers into pursuing the “low lying fruit” and doing analysis which can be trivially decomposed into a set of jobs that run in parallel with each other [25]. Schematically (using the example of an R script analysing a set of differently-sized data sets) such a batch mode of operation is shown in Figure 1.

For many calculations the number of cores required is much larger than can be allocated onto a single computer. Hence, apart from access to the cloud platform itself, job management software is essential. Job managers will do a variety of tasks to aid the above, in particular submitting the tasks to the allocated VMs, creating appropriately named log files and managing failures of individual VMs during the running of the job. This is not trivial to carry out on Amazon EC2. Software such as StarCluster http://star.mit.edu/cluster/ can do this on EC2 but the configuration of StarCluster is a not an easy task and requires a level of familiarity with shell scripts. As a result the full power of these resources can only be employed by computational biologists with extensive experience of shell-scripting as well as expertise in the software they wish to use. This excludes the large number of individuals without those skills whose research could benefit from access to such resources. Cloud computing platforms represent very large software stacks but surprisingly do not by default include this type of job management software.

Azure provides a set of C# libraries referred to as the Generic Worker (GW) to perform a similar set of tasks as a Job Manager. This provides a framework to write C# software to perform the tasks that a Job Manager can do for tailored set of software. Hence it is possible to develop a bespoke interface for users to manage batch jobs for a particular set of software.

### Azure features and comparison

In this section we will provide a more detailed explanation of the Azure infrastructure with comparisons where possible with the Amazon EC2 service. The reader is referred to Table 2 for comparisons of features at a glance. In particular, we will discuss the computational services they provide, disk space, and their ease of use from the perspective of a typical bioinformatics developer with extensive experience of developing software on a Linux architecture and of a biologist with little or no scripting experience. We will also make comparisons from the perspective of costing.

**Table 2:** Comparison of Cloud Computing Features.

Feature	Microsoft Azure	Amazon EC2
Infrastructure provision	PaaS (Cloud Service) and IaaS (Virtual Machines)	IaaS, also PaaS via Elastic Beanstalk
Job Manager?	Via Generic Worker libraries	Yes.
Operating Systems available	Windows Server 2008 on PaaS Windows and Linux on IaaS	Linux and Windows
Data Storage	Mass store	S3 Storage
MapReduce available?	Yes	Yes
SQL available?	Yes	Yes
Ease of use for Linux developer	Learning curve to get familiar with C#; authentication methods not yet trivial	Provision of excellent tutorials plus extensive community support.
Ease of use for user	GW allows development of tailored tools	Requires experience of scripting or workflow software such as Galaxy or Taverna.

### Computational Services

As noted previously, Azure provides an IaaS and PaaS - which Microsoft refer to, respectively, as Virtual Machines and Cloud services. The IaaS offering allows one to deploy VM's which run either pre-built Windows Server 2008 or Linux images, or to upload one's own customized image. This service is similar to the one offered by other IaaS providers but does not make use of the GW libraries discussed above for job management and hence we will not focus further on it here.

The Azure PaaS provides programmatic access from .NET (including C#), Java, node.js, PHP, Ruby and Python though at present the GW libraries are only available for C# and hence the other languages are largely for data transfer. It is comprised of two “roles”: the Web Role designed for setting up a web-based service and the Worker Role which is designed to run applications in a batch production mode. Frequently these can be in parallel with each other, with a web role passing on tasks to a worker role. Both roles use fixed-configuration VMs that are based on a Windows Server Operating System. The major difference between the roles is that the web role has IIS (Microsoft's web server software) installed on it. VMs (and the roles) can vary from the equivalent of 1 CPU with 760 Mbyte memory and 20 Gbyte disk space running nominally on 1 GHz CPU to 8 CPU with 56 Gbyte memory and 2 Tbyte storage running nominally on a 1.6 GHz CPU.

We note that a MapReduce service is also available on Azure called HDInsight.

### Worker Role

**Figure 2:**
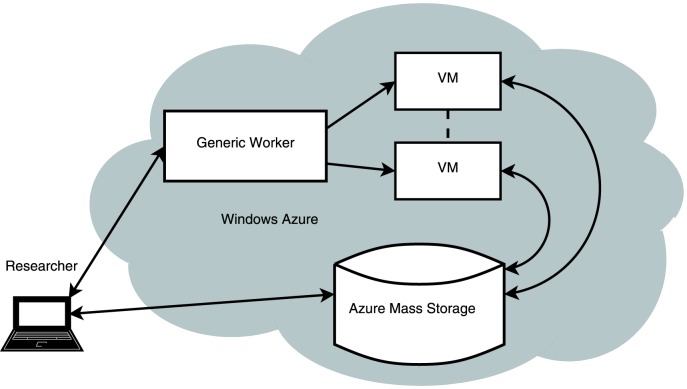
Using Azure with the Generic Worker. Shows that a number of Virtual Machines (VMs) created for the worker roles can be scaled up and down as needed.

The worker role is designed for running large numbers of jobs. Efficient use of this type of role has been improved significantly by the provision of an additional set of libraries from Microsoft called the Generic Worker (GW) which can be accessed from http://resources.venus-c.eu. In particular these libraries can be used within a C# program to submit and efficiently manage jobs submitted to a set of worker roles, as illustrated in Figure 2. In this framework the software runs in two modes. In the first mode an application (which can be a simple executable or a more complex workflow of executables) is uploaded to Azure storage along with a description of the application (in particular the expected list of parameters that will be used in the application). In the second mode the application is transferred from storage and called with a specific set of parameters. The GW provides efficient job management and hence allows a straightforward means to scaling a task. Activation or deactivating of instances of the worker roles can be called within a Windows power shell script or via the code. Additional software can be installed silently at the start of each run. It is possible to construct a workflow where the output from one worker role running one type of executable passes the data onto another worker running another executable and so on, though we have not explored this option. In addition to the demos provided by Microsoft we have developed code that uses the GW to run an R script which can access data that is on Azure storage. The source code for this can be downloaded from http://gene.cs.rhul.ac.uk/RAzure/GWydiR.zip. Details for setting up and using the GW for a sample R script are given in Appendix S1.

### Web Role

As the name suggests the Web Role is designed for setting up web services on Azure. These services can be set up using ASP.NET and C# to create web pages through which a user can interact with programs and data. Web Roles are not designed to run large production runs but can act as a front end by passing data onto worker roles. Obviously this is not optimal for a standard production run where individual failures should be detected and rerun on an as-required rather than as-expected basis. Nonetheless, they can be used to implement tasks such as large uploads of data to the Azure mass storage facilities.

### Data storage and Transfer

Long term storage of data is provided via a mechanism that is similar to Amazon's S3 system. Data is stored in containers which are effectively a single layer of a directory. Individual files are referred to as blobs. It is possible to recreate a pseudo-directory hierarchy by appropriate naming of the blobs with slash characters as in a data path. Microsoft also provides the Azure Marketplace (http://datamarket.azure.com/) where data sets and applications for Azure can be made available.

It is possible to set up a SQL database within Azure for both IaaS and PaaS. The *Azure Storage Explorer* from Neudesic (http://azurestorageexplorer.codeplex.com) allows data to be browsed or transferred to or from Azure storage. We have provided a set of simple Java executables and an R script that enable data transfer to Azure storage within a VM in Azure or on a local machine based on examples provided with Microsoft's documentation. This can be found at https://github.com/hughshanahan/RAzureEssentials.

### Ease of use for Developers

As noted previously, for a developer who is experienced in using Linux systems and is not familiar with a .NET software architecture, designing software using the GW libraries can represent a steep learning curve. On the other hand for a batch mode submission there are templates that can make this substantially easier. The source code for the package corresponds to roughly 400 lines of code, much of which can be taken from the templates available from http://resources.venus-c.eu and the GWydiR github site. As one can see from the walk through for installing the software in Appendix S1, getting initially configured is still not trivial though there is no technical impediment to this being made substantially easier.

### Ease of use for users

From the perspective of the user the same issue of initial configuration is a stumbling block. On the other hand the web resources for managing the VM's and data storage being used are excellent and the user is able to inspect results from the runs via a web interface. Because of the bespoke nature of this it is possible to create an interface that is highly tailored to a specific task and could be substantially easier to use than generic workflow software such as Galaxy and Taverna [26].

### Cost

In Table 3 we provide a comparison of costings between Azure and Amazon EC2. We are not quite comparing like with like in that the pricing for Amazon is using the Linux OS while Azure is using Windows Server 2008 but we are focussing on the cheapest possible option in all cases. We note that pricing can be highly dynamic - for example earlier on in 2013 prices for CPU time on the Azure PaaS were twice that of Amazon EC2. Despite this we can see that market forces influence prices to be roughly comparable (i.e. within the same order of magnitude).

**Table 3:** Cost of some features of Azure and Amazon Cloud Computing.

Feature	Microsoft Azure	Amazon EC2
VM (Small Instance)	$ 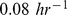 PaaS – Windows	$ 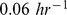 U.S. East - Linux
	$ 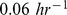 IaaS - Linux	
Ingress	Nothing	Nothing (from Internet)
Egress	$ 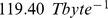	$ 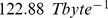
Storage	$ 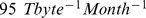 (Mass storage - Globally redundant)	$ 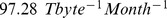 (S3 Standard)

## Materials and Methods

In this work we focus on a real world example to demonstrate the use of R in Azure, namely how G-stacks (probes with runs of 4 or more guanine bases) bias the experiment data for the Affymetrix Human GeneChip called HG_U133A. This GeneChip was studied with a wide scale analysis both because much data is publicly available and because it has the highest ratio of G-stack probes among the Affymetrix Human GeneChips available. It is more beneficial to bioinformaticians to use real data and a useful study than to use an artificial example to evaluate the use of R for bioinformatics in Azure.

The data for many microarray experiments that utilise the HG_U133A GeneChip are available at public repositories such as NCBI Gene Expression Omnibus (GEO) [27], and the EBI ArrayExpress [28]. Each experiment or data set consists of a set of measurements that are stored in CEL files, which can be either binary or character text, depending on the choices of the researcher. We used the data from 576 HG_U133A experiments that were deposited before May, 2012.

The HG_U133A GeneChip contains 22,283 annotated probe sets and about one third of these contain one or more probes with a G-stack in them. Our analysis compared normalised expression values of all probe sets with normalised expression values of probe sets with G-stacks removed. We previously predicted that because probes containing runs of guanine are systematically correlated with each other [29], due to the coherent formation of G-stacks [30], then the difference in normalised expression data between the two sets of results will show a bias. Our analysis also compared correlations of each probe set of the two groups with every other probe set.

The analysis that was carried out on six data sets in GEO [29] on a locally-based computer was repeated on the Azure cloud. The results computed on Azure were the same as those computed locally, hence we are confident of reproducibility. The full analysis on the 576 experiments was performed on Azure. In this paper we will focus on timings, scale and load. Date and time stamp output was written to a log at the beginning and end of loading each set of CEL files, i.e. transferring the files from Azure storage to R working storage. Similar date and time stamp output was also written to the log at the beginning and end of performing the main normalisation and G-stack comparison analyses.

## Results

It is important to understand how the run times on Azure compare with a locally-run calculation. In addition to the time taken to run the script on Azure there is the additional issue of loading the data from the mass storage to the VM. We consider each of these elements in turn.

### Load time

The uploading of the publicly available experiment datasets to Azure mass storage was achieved in two ways. The first method was to use a customised webpage that was initiated and processed by an Azure Webrole (discussed previously). With this method a list of datasets could be passed to the uploading routine. The second method used to upload a few individual experiments was the *Azure Storage Explorer* from Neudesic (mentioned above). This provides a direct link between Azure mass storage accounts and the user's local machine. It can be used to examine files and data in Azure storage and to upload or download individual files. It was less useful when long lists of datasets needed to be uploaded.

**Figure 3:**
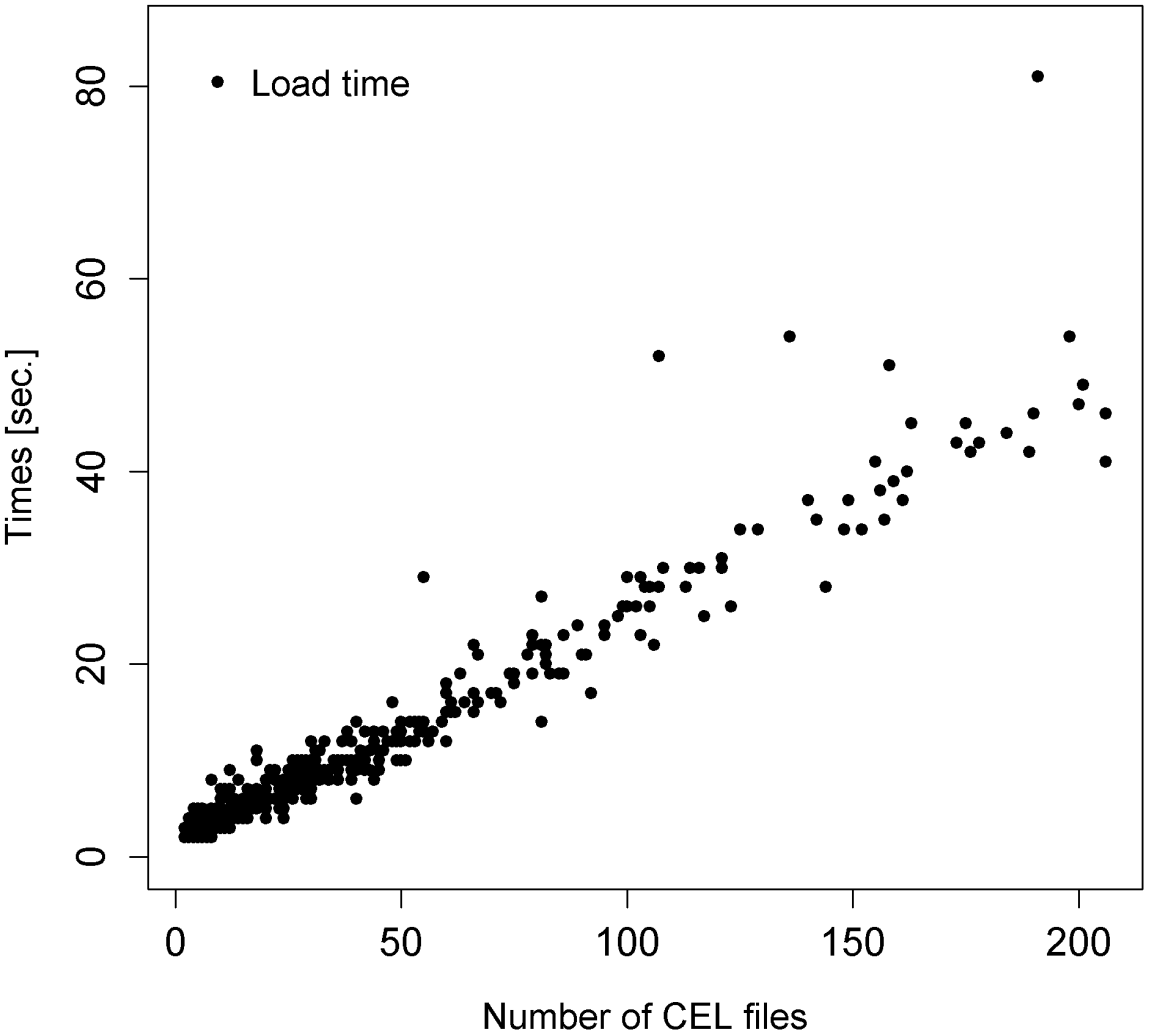
Time taken to load microarray data from Azure mass storage to R working storage. Plot showing the time in seconds taken to load each of 576 datasets from Azure blob storage to local VM disk space, in terms of the number of CEL files in each GSE experiment.

The timings for loading data files from Azure mass storage to R working storage are shown for all 576 HG_U133A experiments in Figure 3. The elapsed time for loading an experiment comprising 2 CEL files (about 23 KBytes for text CEL files) was typically about 2 seconds, and for an experiment comprising 200 CEL files (about 2.25 MBytes for text CEL files) was around 45 seconds. CEL files can be stored in either a text-based or binary format with the text-based format clearly requiring more space. The size of particular CEL files also varies on other factors; for example, how many masks and/or biological outliers the researcher has chosen to record after the intensity data for the array. The outlier experiments in Figure 3 depend on whether the CEL files were stored in binary (shorter load times than the trend) or text-based (longer run times than the trend). The largest outlier had a combination of these formats.

### Run time

**Figure 4:**
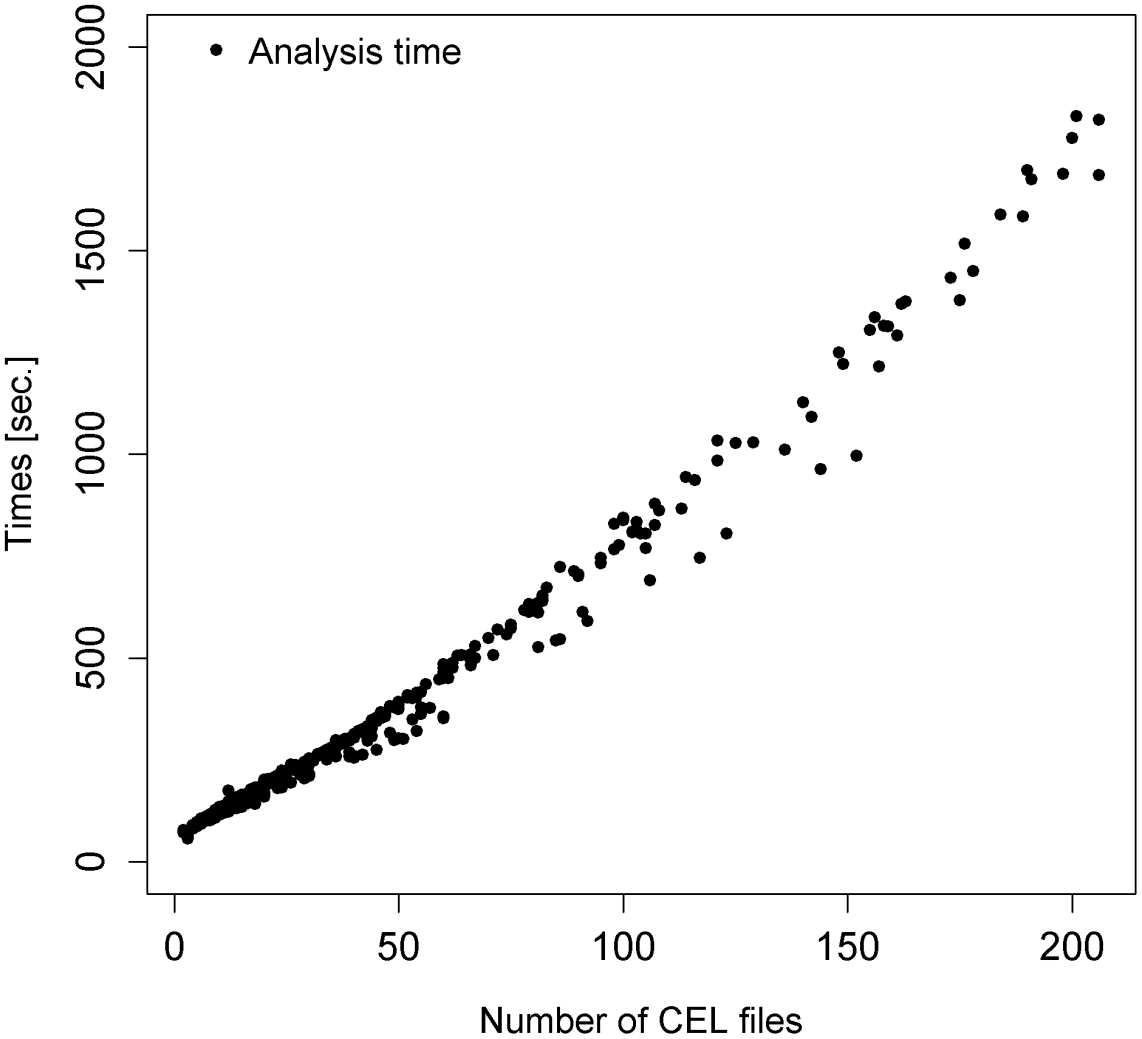
Time taken to analyse data with R script. Shows the time in seconds taken to analyse each of 576 datasets, in terms of the number of CEL files in each GSE experiment.

The timings of the 576 analysis runs (i.e. how long the R scripts ran on an individual VM once the data was loaded) are shown in Figure 4. The outliers below the trend of the data are experiments with binary format CEL files, which are a little quicker to process than the text-format ones.

Once the GW software had been set up and tested, it was a simple matter to scale up the number of VMs to run these analysis jobs. Each experiment was submitted as a separate job. Earlier in our use of Azure we had submitted lists of experiments for analysis runs. It was found that the list approach was less easy to control and scale because sometimes an experiment within the list would fail through a shortage of disk space. By starting each experiment as a new job with fresh disk space this problem was minimised.

### Comparison with using R in a local machine

A set of experiments was chosen to repeat the analyses, which had been done on the Azure cloud, on local machines. The experiments had a range of numbers of CEL files to ensure they were representative of different lengths of jobs. In particular, they had 4, 8, 16, 32, 64 and 128 CEL files.

As it is difficult to reproduce exactly the configuration of an Azure VM, a variety of different local computers were used: -

- Local1 has a 2.13 GHz processor.
- Local2 has a 2.24 GHz processor that runs Windows as a virtual machine.
- As Local2 is run as a virtual machine it is also run with a 70% execution cap to crudely reproduce the nominal VM processor frequency.

**Figure 5:**
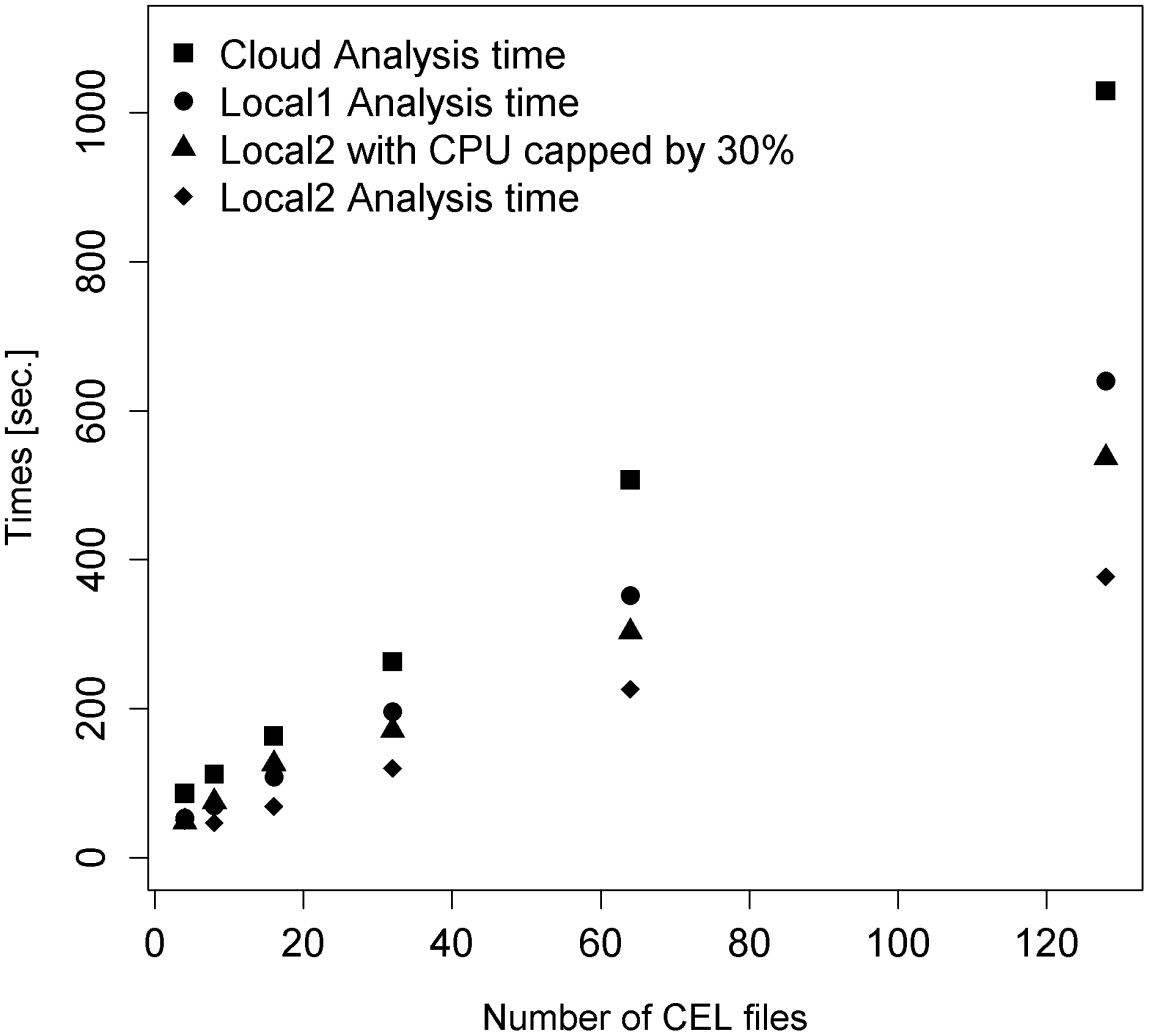
Comparison of Analysis Times between Cloud and 2 Local machines. Shows the time in seconds taken to analyse each of 6 particular experiments, in terms of the number of CEL files in each experiment. The particular experiments were chosen because they had 4, 8, 16, 32, 64 and 128 CEL files, to give a range of experiment data amounts. The machine labelled Local1 had a CPU clock speed of 2.13% CPU cap was added to the Local2 machine to crudely estimate the slower 1.60 GHz stated clock speed of the Azure VM.

The results are shown in Figure 5. In all cases the Azure VM runs more slowly than the local machines, taking roughly a factor of two times as long as the slowest local case.

## Discussion

The wide-spread adoption of cloud computing platforms within bioinformatics has made a major impact on the capability of researchers whose work intermittently requires large amounts of CPU time (or simply large memory) for tasks which can be carried out in a trivially parallel way. The cloud computing paradigm will be of increasing importance for users, particularly as data sets continue to expand in size and hence the analysis will have to come to the data and not the other way around. Up to this point, the majority of bioinformaticians who use cloud computing have made use of Amazon's EC2. It provides a stable software stack with an associated large community of users who can provide support and solutions specific to a researcher's domain. Nonetheless, it is clear that Amazon is no longer the only possible provider of cloud-based solutions and that other approaches should be explored.

In this paper we have specifically examined Microsoft's Azure platform, but we note that many other alternatives exist. The utility of all of these approaches should be considered, if only to ensure that each of the commercial providers remain under pressure to provide as economical a solution as possible.

We have noted that the PaaS infrastructure provided by Azure allows one to develop bespoke interfaces for specific executables that run in a batch mode. Despite the learning curve for developers who do not have a background in writing C# code, these can be put together with a little effort requiring approximately 400 lines of code, much of which can be appropriated from templates. The initial configuration is complex but this could be fixed at first by those developing the bioinformatics pipelines though it is clear that the process could be streamlined further by Azure developers in later releases. In this light, and given the fact that the Worker roles can only run the Windows Server OS it is apparent that the present offering is not a viable solution for bioinformatics software with analyses which are still being developed. On the other hand a substantial set of stable bioinformatics software such as that available via EMBOSS or BioLinux could be deployed very successfully using Azure and its PaaS.

We have shown that the pricing of Azure is comparable with other clouds (at least to within an order of magnitude) though this is highly dynamic. We have also shown that results generated locally are reproduced by equivalent Azure runs and that performance is not substantially affected. Run times suggest that Azure is slower by roughly a factor of 2–3 than local PCs though one has to be careful since we were not able to make a like-for-like comparison using precisely the same CPU-type, memory and exact version of the Windows Operating System.

We have not discussed the general upload of public data as this is a global issue for any public cloud. In tests on a University network we estimated that uploading 1 Tbyte would take approximately 26 hours. This is a rough estimate and is highly dependent on the network connection. However, it is clear that at the very least the large amounts of publicly available data should not be uploaded on an individual basis. The cloud providers that can support this will have a substantial advantage over their competitors. We note that Amazon have made steps in this direction by providing a number of relevant datasets available free of charge such as the data from the modENCODE project [31].

Looking forward the continued blurring between IaaS and PaaS will enable developers and users to make use of the best features of both. Developers can port images created locally to a cloud with precisely the configuration they require, while still being able to run them programmatically so that scaling can be achieved easily with an intuitive interface. If we draw an analogy with web-development, it is possible to imagine an equivalent of Ruby-on-Rails [32] and Django [33]. Both of these enable the easy development of dynamic web sites to be started, that can run using different web server technologies and the underlying scripting languages of Ruby and Python. In the same way one can envisage a similar framework allowing specific executables to be deployed on a cloud (not fixed to any one vendor) and which are run in a batch mode and have an easy interface.

## Supporting Information

Appendix S1 **Scaling an R script on Azure using the Generic Worker.**
(PDF)
