# Supplementary material for: Bioinformatics on the Cloud Computing Platform Azure
Source: PLoS One. 2014 Jul 22;9(7):e102642. doi: 10.1371/journal.pone.0102642 (PMC4106841; doi:10.1371/journal.pone.0102642)
Supplement: Appendix S1 — Scaling an R script on Azure using the Generic Worker. (PDF) [file pone.0102642.s001.pdf]

Appendix : Scaling an R script on Azure using the Generic Worker

This appendix describes how one can make use of Azure and GW libraries to run an R script on multiple data sets that are stored on the Azure mass storage facilities. In particular, this model assumes that the R script is to run individually on the data stored in a container (equivalent of a directory) and that there are an arbitrary number of containers to analyse.

Preliminaries

You will need

- a computer running Windows 7,
- a proficient level of experience running R,
- a text editor such as `notepad++` ,
- a CHM document viewer such SumatraPDF (`http://blog.kowalczyk.info/software/sumatrapdf/free-pdf-reader.html`)
- (optional) Visual Studio 2010.

Setting up Azure account

1. Before using Azure, you will need a Windows (formerly known as a Windows Live) account. This can be easily acquired from the Azure registration page.
2. An account on Azure can be set up by going to `http://www.windowsazure.com/`, clicking on "portal" on the top right hand side of the page and following the instructions. It is also possible to set up a 90 day free usage account from this page which will give more limited resources (e.g. small instance VM's only).
3. Upon completion of registration your browser will be at the Management Portal.
4. You will need to set up
    - a cloud service, which enables deployment of a number of Worker Role VM's to run the script.
    - two container storage areas, one for the data to be stored and one for the application and log files.
5. On the bottom left hand side click on the icon labelled "NEW".
6. From the menu that appears click on "COMPUTE" → "CLOUD SERVICE" (**NOT** "VIRTUAL SERVICE") → "QUICK CREATE". In the box marked "URL" type in the name you want to give to your cloud service. For the purposes of this appendix we will call it `rgwtester`. If you have set up an affinity group select it here or choose a region.

7. Click on the "NEW" icon and then select "DATA SERVICES" → "STORAGE" → "QUICK CREATE". In the "URL" box give it the name of the container storage you want for the data. Here we will call it `rgwdata`.
8. Repeat the previous step to create container storage for the applications, which we will call here `rgwapp`.
9. Clicking on "ALL ITEMS" on the top left hand side of the screen will list the three services you have created.
10. In order to read and write to the storage accounts, you will need their unique primary keys. These can be accessed by clicking on the relevant storage account from the main page, clicking on "MANAGE KEYS" on the centre bottom of the page and copying the long string in the "PRIMARY ACCESS KEY" box.
11. Using your editor, create a text file which on the first line has the name of the container storage for data and on the second has its primary access key. For the above example this file will look like the following

    ```
    rgwdata
    Y4Pn2.....
    ```

    Store it under the name `rgwdata.key`.
12. Do the same for the application container storage. In our case, we will save this file as `rgwapp.key`.

Service package

In order to use your cloud service there are a set of files that must be created and uploaded to your Azure account. Documentation and relevant source files are freely available from `http://resources.venus-c.eu`. We note that all the relevant software for creating the GW libraries can be accessed here, though we will not discuss their compilation.

1. From `http://resources.venus-c.eu/gw/cloud` download the file `ServiceConfiguration.cscfg` and one of the `cspkg` files. The latter set of files determines the size of the VM that you are going to use. For example `Cloud_small.cspkg` is for a small VM with one core and so on.
2. Download and install the latest version of the `STSInitializerSetup` package from `http://resources.venus-c.eu/gw/installers/tools/`.
3. Download and open the documentation file from `http://resources.venus-c.eu/gw/docs/`. In the "Deployment Tutorial" you will find instructions to install the relevant files. The following notes may be of additional use.
    - Item 1 of the tutorial is about making security certificates. You will need to create two certificates. When installing the certificates to your cloud service you will need to upload the certificate (`.cer`) and `.pfx` files.

- In the notes about Creating Service Configuration it is helpful to make use of the Service Configuration Modifier utility (the `STSInitializerSetup` package).
- The instance count in this installer refers to the number of VM's you want running.
- The Data Connection String makes use of the key data for the container storage you use to store your application data.
- The STS thumbprint is the thumbprint of one of the certificates you have created. If you have followed the above instructions on the installation of the certificates you can find them by clicking on the cloud service from the management portal and then selecting the "certificates" option.
- Likewise, the encrypted password can be the same thumbprint.
- The options `AllowInsecure` and `EnableAccounting` should both be set to `TRUE`.
- The service name should be the same as the cloud service (in our example `rgwtest`.
- Likewise in our example the deployment URL should be `http://rgwtest.cloudapp.net`.
- Finally, the Subscription ID can be found by going to the management portal and selecting one of the container storage sites. The ID will be listed on the lower right hand side.
- The final step of the deployment tutorial of the above documentation should not be carried out.

Uploading data

There are a number of different methods to transfer data that we wish to analyse to container storage. For small numbers of files one can use the Azure Storage Explorer which is a simple GUI for inspecting, uploading and downloading containers. This can be downloaded from `http://azurestorageexplorer.codeplex.com/`. For larger numbers of directories we have provided a set of java classes run from the command line which can be downloaded from `https://github.com/hughshanahan/RAzureEssentials`. The R script `uploadDirectories.R` contains a function for uploading a set of directories using these classes.

Running the R script

The cloud service is now ready to accept jobs. Note - when not using the service one should delete the production deployment as Microsoft charges on the basis of whether the service is active, not on whether it is being utillised. For this reason, it is worth uploading your service package file to container storage as this will be deployed much more quickly.

1. Download `http://gene.cs.rhul.ac.uk/Razure/GWydiR.zip`. Unzip and install the package. The source code for this package can be downloaded as a Visual Studio project from `http://gene.cs.rhul.ac.uk/RAure/RAzureSource.zip`.

2. If you have not done so, download the repository `https://github.com/hughshanahan/RAzureEssentials`. In this directory you will find the example R script `exampleScript.R` which can be used as a template. Note :- all of the output log files are stored in container storage so it is not necessary to explicitly upload the log files using the `copy.to.storage` function.

3. A parameters file must also be written. A template file `params.txt` can be found in this directory as well as an explanation.

4. From your start menu run `GWydiR`. A command window will open. Type in the full path for the parameters file you have written (this file name will be kept for future runs). The program will then submit one job for each work item to be analysed.

5. You can inspect the progress of your jobs by going to `http://<yourapp>.cloudapp.net` and clicking on the job management option. The user name and password should be"anonymous" and "researcher".

6. An individual log file will be generated for each item submitted. This is stored in the application container storage in the container `testcontainer`.
